# The Complexities of Kratom: Insights on an Increasingly Frequent Clinical Encounter

**DOI:** 10.7759/cureus.59650

**Published:** 2024-05-04

**Authors:** Adam Alghalith, Hoanglong Nguyen, Richard Tennant

**Affiliations:** 1 Psychiatry, David Geffen School of Medicine, University of California Los Angeles, Los Angeles, USA; 2 Internal Medicine, Olive View-University of California Los Angeles Medical Center, Los Angeles, USA

**Keywords:** drug interactions, withdrawal, opioid, buprenorphine, kratom

## Abstract

*Mitragyna speciosa* (kratom) is a tropical tree native to Southeast Asia with dose-dependent stimulant and opioid properties. Kratom has garnered attention due to its increasing popularity and potential for dependence, tolerance, and withdrawal. We report the case of a 72-year-old man admitted to the hospital for a deep vein thrombosis and obstructive uropathy who began experiencing kratom withdrawal. He experienced less cravings and improvement in withdrawal symptoms with buprenorphine-naloxone 8 mg/2 mg daily. Notably, our case highlights kratom's contributions to a medically complex presentation and potential kratom-drug interactions due to its posited inhibitory effect on cytochrome P450 enzymes and P-glycoprotein. Our case adds to the growing literature which describes buprenorphine-naloxone as an effective treatment for kratom dependence and withdrawal.

## Introduction

*Mitragyna speciosa* (kratom) is a tropical tree native to Southeast Asia [1]. Kratom exhibits stimulant effects at lower doses and opioid effects at higher doses. Kratom leaves contain over 20 alkaloid compounds which are thought to exert psychoactive effects. Mitragynine is the most abundant alkaloid within kratom, and 7-hydroxymitragynine is of interest given its posited effects on opioid receptors. These alkaloids likely act via partial agonism of the mu-opioid receptor, competitive antagonism of the kappa and delta opioid receptors, as well as interactions with serotonergic, adrenergic, and dopaminergic receptors [2,3].

In recent years, kratom has garnered increasing attention due to its popularity and risk of toxicity [4,5]. Kratom is easily accessible legally as an herbal substance, sold on the internet and in gas stations, smoke shops, and corner stores. One study estimates the annual prevalence of kratom use in the United States to be 1.3%, which is comparable to the annual prevalence of lysergic acid diethylamide use [6]. From 2011 to 2017, the annual number of phone calls to the US poison control centers regarding kratom use increased from 13 to 682 [7].

There is a growing literature which describes dependence, tolerance, and withdrawal from regular kratom use. Given its agonism of the mu-opioid receptor, kratom likely parallels the dependence and tolerance induced by more familiar opioids. Kratom is often sold and used as a treatment for opioid withdrawal symptoms, reflecting the similarity between kratom and opioids. Kratom withdrawal symptoms are like those of opioids, including diaphoresis, restlessness, myalgia, lacrimation, diarrhea, tremor, and anxiety [1]. However, more research on kratom dependence is required given its unique mechanism of action and dose-dependent stimulant and opioid effects. Case reports suggest that buprenorphine, a partial opioid agonist, can be utilized for withdrawal symptoms and for long-term maintenance therapy for kratom use disorder (KUD) [8]. However, there are conflicting reports about the dosage of buprenorphine for induction, maintenance, and its correlation to the amount of kratom use [9]. Buprenorphine 4 mg has been effective for withdrawal and dependence; however, daily doses of 24 mg have been used for patients with comorbid pain, depression and anxiety, or concomitant opioid use [10-13].

Kratom also likely affects the metabolism of other drugs via the inhibition of cytochrome P450 (CYP) enzymes 1A2, 2C19, 2D6, 2C9, and 3A4 and the inhibition of P-glycoprotein (P-gp) [14,15]. In vitro studies suggest concentration-dependent inhibition of the major CYP enzymes, specifically 2D6, 2C9, and 3A4. This set of CYP enzymes is involved in the metabolism of an extensive number of medications including antibiotics, statins, anticoagulants, antiarrhythmics, antipsychotics, antidepressants, and antihypertensives [16]. Consideration should also be given to the chemical composition of kratom as studies have suggested that the wide variability of kratom products can affect its opioid receptor activity and interactions with CYP enzymes [14].

Our case report supports the use of buprenorphine for kratom withdrawals and dependence. It also demonstrates the potential adverse side effects of kratom, which are like opioids given kratom's mechanism of action. Our medically complex case also calls attention to potentially harmful kratom-drug interactions.

## Case presentation

A 72-year-old man with a history of benign prostatic hyperplasia, hypertension, and hyperlipidemia presented to the emergency room with worsening bilateral leg swelling over four months.

On presentation, he was noted to have an oxygen saturation of 97% without tachycardia or tachypnea. His medications included losartan and hydrochlorothiazide for hypertension, tadalafil for erectile dysfunction, testosterone injections approximately every two weeks for low libido, and quetiapine for insomnia. Physical exam revealed bibasilar inspiratory crackles, an unremarkable cardiac exam, and an abdominal exam notable for soft suprapubic distension with tenderness. Notably, he had bilateral lower extremity edema with left calf tenderness. Lab values (Table 1) were notable for a hemoglobin of 9.7 mg/dL, hematocrit of 29.4%, and creatinine of 1.9, elevated over his known baseline of 1.4, indicating an acute kidney injury possibly superimposed on chronic kidney injury. He had mild normocytic anemia with an iron panel indicative of chronic disease or chronic kidney damage although his baseline is not known.

**Table 1:** Admission laboratory testing CBC: complete blood count; WBC: white blood cell; RBC: red blood cell; Hgb: hemoglobin; Hct: hematocrit; MCV: mean corpuscular volume; Na: sodium; K: potassium; Cl: chloride; CO2: carbon dioxide; BUN: blood urea nitrogen; Cr: creatinine; Ca: calcium; Mg: magnesium; Alk Phos: alkaline phosphatase; AST: aspartate aminotransferase; ALT: alanine aminotransferase; TIBC: total iron-binding capacity; TSH: thyroid-stimulating hormone; Hgb A1C: hemoglobin A1C; PSA: prostate-specific antigen

Lab	Value	Reference range
CBC		
WBC	7	4.5-10 x 10^3^/uL
RBC	3.22	4.4-5.6 x 10^6^/uL
Hgb	9.7	13.5-16.5 g/dL
Hct	29.40%	40-49%
MCV	91.3	82-97 fL
Platelets	258	160-360 x 10^3^/uL
Basic metabolic panel		
Na	138	136-144 mmol/L
K	3.9	3.6-5.1 mmol/L
Cl	101	97-108 mmol/L
CO2	29	22-32 mmol/L
BUN	24	8-20 mg/dL
Cr	1.99	0.5-1.2 mg/dL
Glucose	96	65-139 mg/dL
Electrolytes		
Ca	8.8	8.9-10.3 mg/dL
Mg	2	1.8-2.5 mg/dL
Liver function tests		
Albumin	4.1	3.5-4.8 g/dL
Alk Phos	49	38-126 U/L
AST	20	15-41 U/L
ALT	21	14-54 U/L
Total bilirubin	0.9	0.1-1.2 mg/dL
Iron studies		
Iron	85	37-164 ug/dL
TIBC	256	261-478 ug/dL
Iron saturation	33.20%	N/A
Ferritin	359	22-275 ng/mL
Transferrin	183	180-382 mg/dL
Vitamins		
Folate	4.3	>7 ng/mL
B12	397	213-816 pg/mL
Thyroid function tests		
TSH	1.033	0.35-4.94 uIU/mL
Free T4	1	0.7-1.48 ng/dL
Others		
Hgb A1C	5.70%	<5.6%
PSA total	6.27	<4 ng/mL

A lower extremity venous duplex ultrasound revealed a nearly occlusive thrombus of the left mid to distal peroneal vein. Due to his elevated creatinine and history of lower urinary tract symptoms, the patient also received a bedside ultrasound and confirmatory renal ultrasound suggesting bilateral hydronephrosis and prostatomegaly. After placing a Foley catheter and starting the patient on heparin, he was admitted to medicine for the management of obstructive uropathy and deep vein thrombosis (DVT).

On day 2 of his hospitalization, the patient started experiencing mild withdrawal symptoms, including diaphoresis, loose stools, tremulousness, yawning, and anxiety, with a Clinical Opiate Withdrawal Scale (COWS) score of 9 (Table 2). He revealed that he had been using kratom for 10 years as an herbal supplement to address pain or stress in his daily life. He had not experienced withdrawal symptoms in the past, so he was not cognizant of a potential dependence on kratom, although he admits to never being able to self-wean off kratom. Given the opioid activity of kratom, the patient was started on clonidine 0.2 mg three times daily as needed and Ativan 0.5 mg four times daily as needed for his withdrawal symptoms. The addiction medicine service recommended to start him on buprenorphine-naloxone 4 mg/1 mg once daily given recent case reports on its effectiveness for kratom withdrawal and dependence. The patient still complained of mitigated but persistent diaphoresis and tremulousness, prompting an increase in his buprenorphine-naloxone to 8 mg/2 mg once daily which controlled his withdrawal symptoms, reflected by a COWS score of 0. During hospitalization, he was transitioned from heparin to rivaroxaban and noted to have improved kidney function without signs of post-obstructive diuresis after Foley removal. The patient was discharged on buprenorphine-naloxone 8 mg/2 mg once daily.

**Table 2:** COWS on hospital day 2 COWS: Clinical Opiate Withdrawal Scale; GI: gastrointestinal

Symptom	Score
Resting pulse rate	0
Sweating	2
Restlessness	0
Pupil size	0
Bone or joint aches	0
Runny nose or tearing	0
GI upset	2
Tremor	2
Yawning	2
Anxiety or irritability	1
Gooseflesh skin	0
Total score	9

## Discussion

Our case further corroborates the use of buprenorphine for KUD. The patient recognized his kratom dependence on hospital day 2 when he started experiencing withdrawal symptoms. Our patient experienced relief from withdrawal symptoms after escalating doses of buprenorphine from 4 mg to 8 mg daily, which is in line with induction and maintenance dosing reported in the literature [11]. This dose of buprenorphine suggests that kratom induces a relatively large degree of tolerance. To put this in context, buprenorphine 8 mg to 16 mg is typically prescribed as medication-assisted treatment for individuals who use heroin. For those who use fentanyl, buprenorphine doses can range from 24 mg to 32 mg. And in cases of chronic pain or cancer pain, buprenorphine is often prescribed in the sub-milligram range [17,18]. We have found that buprenorphine can be a useful medication in treating cravings and withdrawal from kratom as part of medication-assisted treatment. Several induction methods of buprenorphine have been reported to be successful in controlling cravings and withdrawal. However, maintenance dosing is less clear and varies widely based on comorbid pain, depression and anxiety, or concomitant opioid use [10-13]. There is also little evidence of eventual tapering of buprenorphine. To guide medication-assisted treatment and prevent relapse, more research is needed to establish maintenance dosing and tapering of buprenorphine in KUD. The widely varying chemical composition of kratom products should also be acknowledged as a source of variability in the degree of drug tolerance and the consequent dose of buprenorphine required [14].

Kratom use likely contributed to his complex presentation and affected his treatment plan. The patient's obstructive uropathy was most likely due to his benign prostatic hyperplasia with smaller contributions from regular kratom use. Kratom use can hypothetically result in urinary retention via kratom's activation of mu-opioid receptors, leading to the inhibition of parasympathetic nerves which impact detrusor muscles and sphincter tone. The patient's DVT etiology is not entirely understood, and while it was thought to be attributed to his regular testosterone injections, he did not have erythrocytosis which is the proposed mechanism leading to DVT. However, there are patients on testosterone who do develop DVT without erythrocytosis due to other mechanisms related to the dose of testosterone [19]. These alternative mechanisms raise concern over the possibility of kratom's inhibition of testosterone metabolism from CYP 3A4 and thus contribute to these other mechanisms leading to DVT [20].

Lastly, our patient used other medications with metabolism potentially affected by kratom use. He presented with prescriptions for losartan, hydrochlorothiazide, and quetiapine. There are noted cases of quetiapine-kratom drug interactions since quetiapine is a CYP 3A substrate; however, our patient did not present with signs of quetiapine toxicity including QTc abnormalities [15]. More research is required to elucidate the extent of kratom's effects on drug metabolism, specifically future clinical studies as there are several in vivo studies of kratom [14]. Regardless, clinicians should be aware of kratom's potential interactions with sedatives, resulting in oversedation, and CYP substrates, such as antipsychotics, antihypertensives, and anticoagulants [16].

Providers should be cognizant of the increasing prevalence of kratom use and its implications in clinical practice. Many patients, including the patient in this report, view kratom as a harmless herbal supplement. When asking patients about substance use, we recommend that providers ask specifically about kratom use since it is easily accessible on the internet and in smoke shops. Patients should be counseled on kratom's dose-dependent stimulant and opioid activity, its potential for drug-drug interactions, and its side effects. Clinicians should consider buprenorphine or buprenorphine-naloxone to address withdrawal and dependence symptoms. Providers should also adjust the dose of buprenorphine according to comorbidities and increased tolerance from concomitant opioid use.

## Conclusions

Kratom is an increasingly popular substance with dose-dependent stimulant and opioid properties. Buprenorphine can be an effective treatment for kratom dependence and withdrawal, and the dose should be adjusted based on comorbidities and concomitant drug use. Clinicians should consider kratom-drug interactions given its posited inhibitory effect on CYP enzymes and P-gp.
